# Beliefs, perceptions and practices of chiropractors and patients about mitigation strategies for benign adverse events after spinal manipulation therapy

**DOI:** 10.1186/s12998-020-00336-3

**Published:** 2020-09-08

**Authors:** Martha Funabashi, Katherine A. Pohlman, Rachel Goldsworthy, Alex Lee, Anthony Tibbles, Silvano Mior, Greg Kawchuk

**Affiliations:** 1 Department of Chiropractic, Université du Québec à Trois-Rivières, 3351 boul. Des Forges, Trois-Rivières, QC G8Z 4M3 Canada; 2 Canadian Memorial Chiropractic College, 6100 Leslie St, Toronto, ON M2H 3J1 Canada; 3 Parker University, 2540 Walnut Hill Lane, Dallas, TX 75229 USA; 4 University of Alberta, 8205 114 St, Edmonton, AB T6G 2G4 Canada

**Keywords:** Spinal manipulation, Adverse events, Mitigation, Survey, Patient safety, Quality assurance

## Abstract

**Background:**

Approximately 50% of patients who receive spinal manipulative therapy (SMT) experience some kind of adverse event (AE), typically benign and transient in nature. Regardless of their severity, mitigating benign AEs is important to improve patient experience and quality of care. The aim of this study was to identify beliefs, perceptions and practices of chiropractors and patients regarding benign AEs post-SMT and potential strategies to mitigate them.

**Methods:**

Clinicians and patients from two chiropractic teaching clinics were invited to respond to an 11-question survey exploring their beliefs, perceptions and practices regarding benign AEs post-SMT and strategies to mitigate them. Responses were analyzed using descriptive statistics.

**Results:**

A total of 39 clinicians (67% response rate) and 203 patients (82.9% response rate) completed the survey. Most clinicians (97%) believed benign AEs occur, and 82% reported their own patients have experienced one. For patients, 55% reported experiencing benign AEs post-SMT, with the most common symptoms being pain/soreness, headache and stiffness. While most clinicians (61.5%) reported trying a mitigation strategy with their patients, only 21.2% of patients perceived their clinicians had tried any mitigation strategy. Clinicians perceived that patient education is most likely to mitigate benign AEs, followed by soft tissue therapy and/or icing after SMT. Patients perceived stretching was most likely to mitigate benign AEs, followed by education and/or massage.

**Conclusions:**

This is the first study comparing beliefs, perceptions and practices from clinicians and patients regarding benign AEs post-SMT and strategies to mitigate them. This study provides an important step towards identifying the best strategies to improve patient safety and improve quality of care.

## Introduction

Spinal manipulative therapy (SMT) is a manual therapy technique commonly used by many healthcare professionals, including chiropractors, osteopaths, physiotherapists and naprapaths. It is used to treat many musculoskeletal conditions, especially low back and neck pain [1]. Specifically for chiropractors, SMT is the most common treatment provided during a patient encounter [2–4]. It is estimated that the 12-month utilization rate for chiropractic services is 9.1% and a lifetime utilization of 22.2% worldwide [4]. Between 1980 and 2015, the use of chiropractic services has reportedly increased from 10.0 to 11.7% in Canada and from 7.2 to 10.7% in the United States [4].

Similar to most health care interventions, adverse events (AEs) have been observed after SMT. Reported post-SMT AEs vary in terms of frequency and severity, ranging from the more frequent minor/benign AEs (such as increased soreness and stiffness) to rare and serious AEs (such as cauda equina syndrome) [5]. Previous studies estimated that about 50% of patients receiving SMT experience some kind of AE, most being benign, transient and self-resolving with little to no impact on activities of daily living [5–7].

Potential predictors of benign AEs post-SMT have been previously reported [8–12] such as sex, SMT technique, multiple treated locations, working status of patient, and duration of pain at presentation. Although benign AEs may be considered to be an expected consequence of care [13], it remains unknown if benign AEs represent inherent effects resulting from SMT itself, a component of the natural history of the patient’s presenting condition, and/or inadequacies regarding the therapeutic encounter (such as inappropriate technique). Although post-SMT AEs are typically benign and self-limiting, their presence can influence patient perceptions, expectations, well-being and quality of life [14]. Therefore, while it is important to investigate mechanisms underlying benign AEs post-SMT to eliminate their incidence, efforts should also be made concurrently to mitigate AEs so as to improve patients experience and quality of care.

Strategies to mitigate AEs have been widely hypothesized and investigated across health care professions with varying degrees of success [15–17]. For example, the prolonged application of pressure after venepuncture has been observed to reduce the incidence and size of venepuncture-related bruises [15]; warming or rubbing adult diphtheria tetanus vaccine inoculation does not reduce the incidence of pain after administration [16]; and there are no differences in pain perception with the use of a topical skin coolant prior to intravenous catheter placement [17].

Specifically related to SMT, previous studies investigating mitigation strategies have included assessment of risk factors related to serious AEs post-cervical SMT [18, 19]. No study has yet investigated strategies for mitigating benign AEs post-SMT. The first step in this process is to better understand what mitigation strategies are currently used by clinicians, and those perceived by patients, in reducing AEs after SMT. The aim of our study was to identify beliefs, perceptions and practices of chiropractors and patients regarding benign AEs post-SMT and potential strategies to mitigate them.

## Methods

A cross-sectional survey design was used. We surveyed supervising clinicians and patients at two chiropractic teaching institutions. The survey was available electronically between June and August 2019. This study was reviewed and approved by the Research Ethic’s Boards from the Canadian Memorial Chiropractic College (CMCC) (study #1901B04) and Parker University (study #A-00187).

### Clinician sample

The lead investigator at each chiropractic teaching institution invited all supervising clinicians at CMCC (*n* = 35) and Parker University (*n* = 23) to respond to an electronic survey via email (total *n* = 58). Three email reminders as well as personal communication by a research assistant were used to encourage and remind clinicians of the survey.

### Patient sample

A convenience sample of 107 patients at the CMCC’s Campus Clinic and 123 patients at Parker’s Wellness Clinic receiving chiropractic treatment were invited to respond to the online survey. If the patient agreed to participate, they were provided with a tablet and directed to complete the survey in a private quiet area. Patients were provided with both the email address and phone number of the lead investigator to contact if they had any questions. The reason of patients who declined participation at both CMCC and Parker was not recorded.

### Survey questions

Both clinician and patient surveys were developed through an informal process of expert opinion. The clinician survey consisted of 11 questions regarding experiences and beliefs related to benign AEs post-SMT (rating scale: ‘yes’, ‘no’ or ‘don’t know’) and strategies currently used or recommended to mitigate them (rating scale: ‘is possible’, ‘may be possible’, or ‘not possible’). Questions were presented in multiple-choice forma, including an “other” option to collect information not captured by the multiple-choice format. The patient survey consisted of 11 questions regarding experiences with SMT and benign AEs (rating scale: ‘yes’, ‘no’ or ‘don’t know’), as well as strategies perceived to mitigate them (rating scale: ‘is possible’, ‘may be possible’, or ‘not possible’) and strategies their provider may have utilized in the past. Similar to the clinician’s survey, an “other” option was available for each question.

Both surveys also included demographic questions and a fixed list of potential mitigation strategies in which participants were asked to state the probability that each listed strategy could reduce the frequency and/or intensity of benign AEs post-SMT. Prior to distribution, both surveys were validated for content by the research team and pilot tested with practicing chiropractors and patients (*n* = 3 for each survey). The survey was distributed and data collected using Research Electronic Data Capture (REDCap), a secure, web-based application designed to support data capture for research providing an intuitive interface for validated data entry, audit trails for data manipulation and export procedures [20].

### Data analysis

All responses from both institutions were imported into Microsoft Excel spreadsheets, where data was analyzed. For each survey question, the response percentage was calculated for each multiple-choice option. Responses for the outcome of interest were analyzed by institution. Responses from clinicians and patients were also compared to each other.

## Results

### Clinicians

A total of 39 clinicians participated in the study (overall response rate of 67%): 21 from CMCC (60%) and 18 from Parker University (78%). Table 1 presents the characteristics of responding clinicians with most having more than 10 years in practice (61.5%) and between 1 to 5 years as a supervising clinician (53.8%). Overall, most (66.7%) participating clinicians from Parker University had less years as supervising clinicians (1–5 years of practice) than those at CMCC, where 42.9% had 1–5 years as supervising clinicians and 47.6% had more than 10 years. However, most clinicians at both institutions (71% CMCC and 55% Parker University) reported similar number of years in practice (more than 10 years).

**Table 1:** Characteristics of responding clinicians (*n* = 39)

	CMCC, n (%)	Parker, n (%)	TOTAL, n (%)
**Years in practice**			
1–5	1 (4.8)	5 (27.8)	6 (15.4)
5–10	5 (23.8)	4 (22.2)	9 (23.1)
More than 10	15 (71.4)	9 (50.0)	24 (61.5)
**Years as a supervising clinician**			
1–5	9 (42.9)	12 (66.7)	21 (53.8)
5–10	2 (9.5)	1 (5.6)	3 (7.7)
More than 10	10 (47.6)	4 (22.2)	14 (35.9)
**Profession credentials**			
Chiropractic	21 (100)	18 (100)	39 (100)
Athletic therapy	1 (4.8)	1 (5.6)	2 (5.1)
Acupuncture	4 (19.0)	4 (22.2)	8 (20.5)
Masters degree	6 (28.6)	2 (11.1)	8 (20.5)

### Patients

A total of 203 patients (overall response rate of 88.2%) completed the survey at CMCC’s Campus Clinic (*n* = 101; 94.4% response rate) and Parker’s Wellness Clinic (*n* = 102; 82.9% response rate). Characteristics of patients who participated in this study are presented in Table 2. Most patients reported receiving more than 10 SMTs (65%) during their program of care over a period of more than 10 months (56.2%). While most patients from CMCC received more than 10 SMTs (79.2%) and over 10 months of chiropractic treatment (75.2%), Parker University patients’ experience was more diverse with 51% having received more than 10 SMTs and only 37.3% receiving chiropractic care for over 10 months.

**Table 2:** Characteristics of participating patients (*n* = 203)

	CMCC, n (%)	Parker, n (%)	TOTAL, n (%)
**Number of SMT received**			
1–5	14 (13.8)	34 (33.3)	48 (23.6)
5–10	7 (6.9)	13 (12.7)	20 (9.9)
More than 10	80 (79.2)	52 (51.0)	132 (65)
**Months receiving chiropractic care**			
Less than 1 month	10 (9.9)	24 (23.5)	34 (16.7)
1–3 months	8 (7.9)	22 (21.6)	30 (14.8)
3–10 months	7 (6.9)	16 (15.7)	23 (11.3)
More than 10 months	76 (75.2)	38 (37.3)	114 (56.2)
**Other health care professionals being sought**			
Medical Doctor (MD)	25 (24.8)	12 (11.8)	37 (18.2)
Physiotherapist	15 (14.9)	2 (2.0)	17 (8.4)
Athletic Therapist	2 (2.0)	0 (0)	2 (1)
Acupuncturist	7 (6.9)	0 (0)	7 (3.4)
Naturopath	7 (6.9)	1 (1.0)	8 (3.9)
Massage Therapist	41 (40.6)	12 (11.8)	53 (26.1)
None (seeing chiropractor only)	37 (36.3)	70 (68.6)	107 (52.7)

*CMCC* Canadian Memorial Chiropractic College, *SMT* spinal manipulative therapy

### Benign adverse events (AEs)

**Fig. 1:**
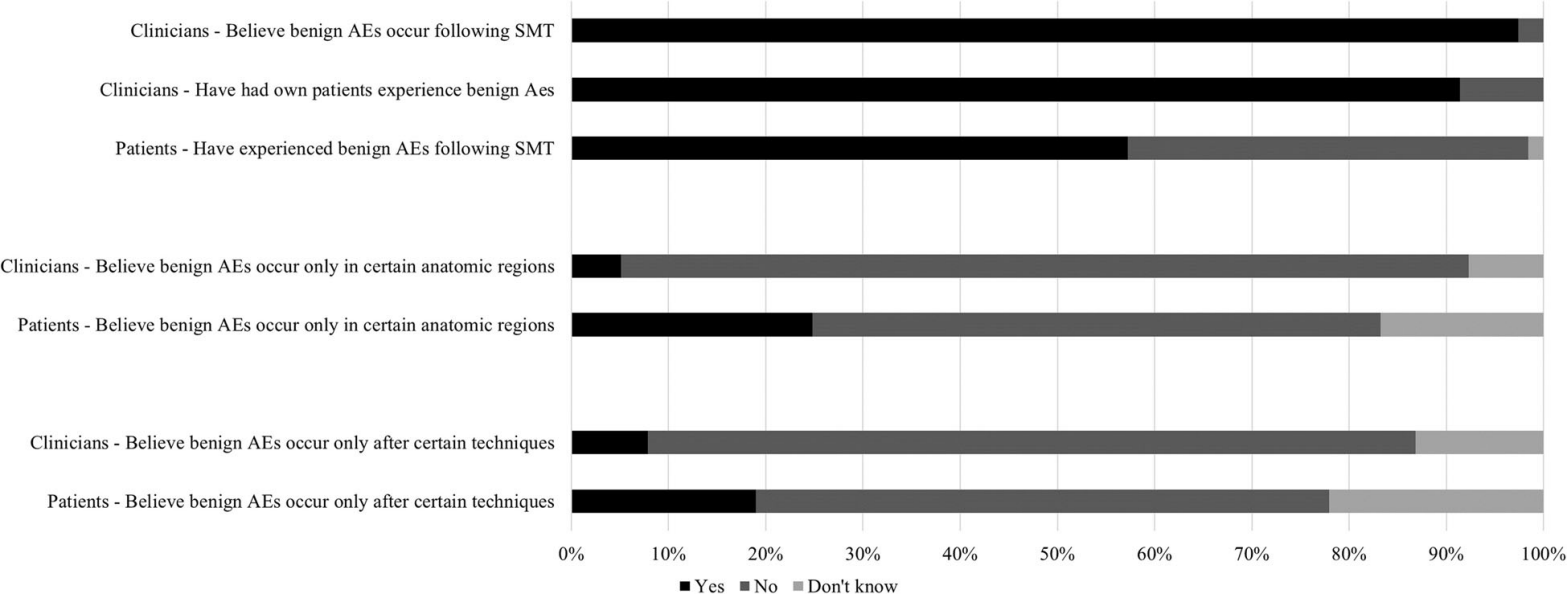
Percentages of clinicians and patients’ perceptions and experiences regarding benign adverse events. Legend: AEs – adverse events; SMT – spinal manipulative therapy

Figure 1 presents perceptions and experiences of clinicians and patients regarding benign AEs post-SMT. Most clinicians believed benign AEs occur post-SMT (97.4%) with 82.1% reporting their own patients having experienced such events. In terms of frequency of occurrence of benign AEs, most clinicians (74.4%) believe they occur infrequently; however, a few clinicians (15.4%) did believe that such events occur “quite often”. Although, the majority of clinicians did not believe benign AEs were related to specific SMT techniques or anatomic region, one (2.6%) did believe benign AEs were related to cervical SMTs.

**Fig. 2:**
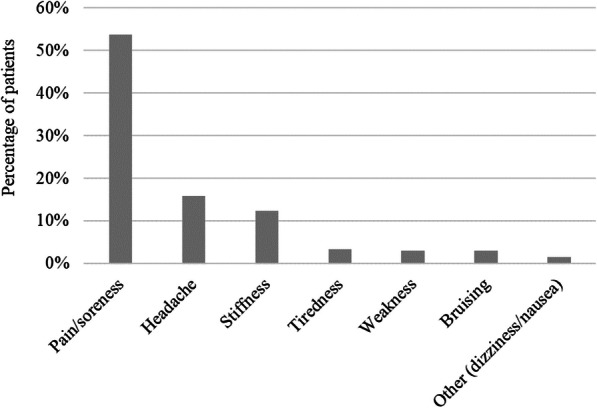
Percentage of participating patients who experienced each type of benign adverse events (*n* = 202)

Most patients (55%) reported experiencing benign AEs post-SMT, of which pain/soreness, headache and stiffness were the most common reported events (Fig. 2). Even though most patients did not believe benign AEs were related to specific SMT techniques or body region, 13 (6.4%) patients believe rotatory techniques specifically to the cervical region are related to benign AEs. Among the 24.1% of patients who do believe benign AEs occur after SMT to a specific body region, 20% believe benign AEs occur after SMT is applied to the cervical region and 5.5% after SMT is applied to the lower back.

### Strategies for mitigating benign AEs

**Fig. 3:**
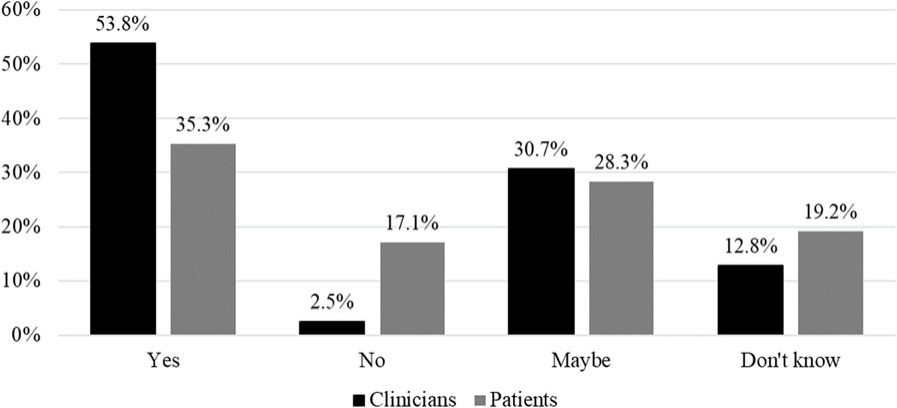
Percentage of clinicians and patients reported beliefs regarding the possibility of mitigating benign adverse events (*n* = 39 clinicians; *n* = 198 patients)

Most clinicians (53.8%) and some patients (35.3%) believe that mitigating benign AEs “is possible”, with an additional 30.7% of clinicians and 28.3% of patients believing it “may be possible”. Over half of the clinicians (61.5%) reported previously trying a mitigation strategy with their patients; yet, only 21.2% of patients believed their clinicians had tried any mitigation strategy (Fig. 3).

From the clinicians reporting using strategies to mitigate benign AEs post-SMT (*n* = 24), the most common strategies were: soft tissue therapy (75%), stretching (62.5%) and icing (45.8%). All 24 clinicians reported success using these strategies to mitigate benign AEs post-SMT, mainly based on patient-reported symptom improvement (69.2%), but also based on palpatory (15.3%) and visual physical (15.3%) changes post intervention. From the clinicians who believe mitigating benign AEs “is possible” (*n* = 21), 95.2% (*n* = 20) have tried a mitigation strategy with the majority (*n* = 15, 75%) having tried soft tissue therapy. Among clinicians who believe mitigating benign AEs “may be possible” (*n* = 12), 33.3% (*n* = 4) reported having tried a mitigation strategy with all of them (*n* = 4, 100%) reporting trying stretching.

In general, patients who reported that their clinicians tried a mitigation strategy (21.2%), 55.8% reported their clinician used soft tissue therapy, 44.1% believed they used stretching and 30.2% believed heat was used. Most patients (97.6%) reported success using strategies to mitigate benign AEs, with 58.1% reporting having no benign AEs after using mitigation strategies and 40.7% reported experiencing benign AEs that were less severe than expected after using the mitigation strategy. Specifically, among patients who believe mitigating benign AEs “is possible” (*n* = 70), 57.2% (*n* = 33) reported that their clinician had tried a mitigation strategy with 18 (54.5%) of them specifying that soft tissue therapy was the applied strategy. Among patients who believe mitigating benign AEs “may be possible” (*n* = 10), 60% (*n* = 6) reported their clinician most commonly used soft tissue therapy as the most common mitigation strategy.

**Fig. 4:**
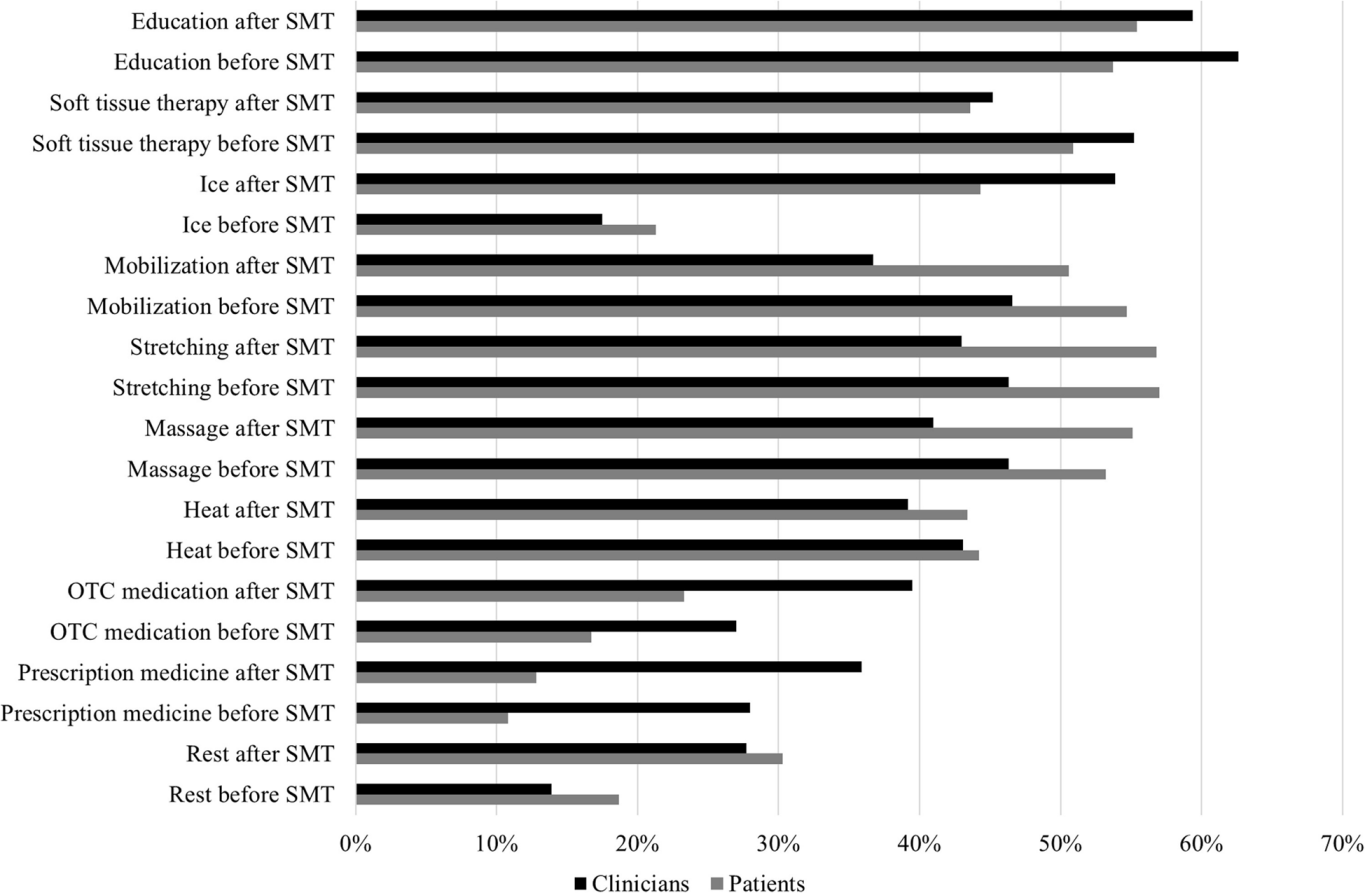
Percentage of clinicians and patients’ beliefs regarding the likelihood of specific mitigation strategies reducing frequency and severity of benign adverse events (AEs) after spinal manipulative therapy (SMT). (*n* = 39 clinicians, *n* = 202 patients). Legend: OTC – over the counter

From the fixed list of potential mitigation strategies included in our survey, Fig. 4 illustrates the likelihood of specific mitigators to reduce the frequency or severity of benign AEs post-SMT as per beliefs and perceptions of participating clinicians and patients. Clinicians perceived that patient education, either before or after treatment, was most likely to mitigate benign AEs post-SMT, followed by soft tissue therapy and/or icing following SMT. Patients perceived stretching, either before or after SMT, was the strategy most likely to mitigate benign AEs from occurring, followed by education and/or massage after SMT. No additional strategy was suggested in the “other” field by clinicians or patients.

## Discussion

This study identified the beliefs, perceptions and practices of clinicians and patients regarding benign AEs post-SMT and strategies to mitigate them. To the best of our knowledge, this is the first study to address this topic. While participating clinicians and patients were aware that benign AEs occurred post-SMT, they perceived different factors related to their occurrence. Both clinicians and patients identified common strategies to mitigate benign AEs, including education. Understanding these beliefs, perceptions and practice of mitigation strategies are important steps towards advancing the investigation of management care plans. By elucidating effective strategies to reduce benign AEs post-SMT, improvement might be expected in patient safety, patient expectations and quality of care.

Interestingly, some responding clinicians and patients reported that benign AEs occur after rotatory SMT techniques performed on the cervical region. This is in accordance with a recent study by Funabashi and Carlesso [21] reporting that patients receiving treatment for neck conditions most frequently perceive the symptoms they experience after manual therapy as AEs. Cervical SMT is often performed in patients with neck conditions and despite the rare frequency of these events and a lack of causal association between cervical SMT and serious AEs (such as stroke and vertebral artery dissection), they receive significant media attention [12, 22–24]. As such, it is possible that the media portrayal and focus on serious AEs following cervical SMT may influence patients’ perception related to AEs associated with cervical SMT. This is an interesting topic and further research investigating how media portrayals influence patients’ perceptions of AEs post-SMT should be conducted.

Previous studies have reported that about 50–60% of patients have benign AEs post-SMT, such as local discomfort and headache [8, 10, 11]. Our findings are in accordance with these reports, with 55% of the patients reported experiencing a benign AEs post-SMT, with soreness and headache being the most common symptoms. These numbers suggest that despite the benign nature of these AEs, their frequency is high and establishing strategies to mitigate them can have a positive impact on patients’ SMT experience, improving their quality of care.

Regarding mitigation strategies, most participating clinicians and patients report mitigation of benign AEs post-SMT is, or may be, possible (Fig. 3). This is encouraging as previous studies have investigated mitigation strategies in other health care areas with similar positive outcomes. For example, cold sprays and topical anesthetics have been observed to decrease pain of intravenous placement in adults and children, respectively [25, 26]. Additionally, phlebotomists ensuring that haemostasis had been attained before leaving the patient was found to significantly reduce the number and size of bruising after venepuncture [15]. Although pain during intravenous catheter placement and bruising after venepuncture are not major AEs that significantly affect patients’ health, strategies to mitigate these symptoms have been investigated in order to improve patient’s experience and quality of service. In a similar way, investigations of strategies to mitigate benign AEs post-SMT should be conducted focusing on approaches identified by clinicians and patients.

Participating clinicians and patients who indicated having previously applied strategies to mitigate benign AEs post-SMT included soft tissue therapy, stretching, ice and heat. The use of these strategies was perceived to be successful by clinicians based on their own patients self-reported improvement, and by patients, based on their own experience (no benign AEs experienced or with reduced severity). Given no studies to date have investigated mitigation strategies for SMT, our data provides important preliminary information of potentially clinically relevant strategies that can be assessed in future investigations. More specifically, prospective investigations assessing the effectiveness of the mitigation strategies identified by clinicians and patients are currently being designed.

Both clinicians and patients perceived that education is the strategy most likely to mitigate benign AEs. Indeed, a previous study emphasized the importance of patient education regarding post-treatment responses and how this can contribute to patients’ perceptions of AEs following manual therapy [14]. More specifically, patients have indicated the importance of receiving education regarding the potential AEs following treatment. Having an informed expectation about potential AEs reassured patients that what they may experience following treatment was acceptable [14, 27].

The discrepancy between the percentage of clinicians who reported previously trying a mitigation strategy and patients who believed their clinician tried a mitigation strategy may be an indication of a potential lack of communication. It is possible that clinicians may not communicate all their reasons for applying specific interventions to patients and while clinicians believe they tried a mitigation strategy, their patients were not aware of it. Due to the anonymous nature of the data, we were unable to directly link the discrete responses of clinicians to their patients and, therefore, cannot verify if clinicians who reported having tried mitigation strategies had their patients participating in this study. Enhancing the communication between patients and clinicians is believed to also enhance patient involvement and participation in monitoring their own health, potentially increasing patient-centered care approach [28]. Future study may shed further light on the impact of communication as a mitigator.

Clinicians and patients also suggested that soft tissue therapy and massage after SMT could be used to mitigate benign AEs. Similarly, participating patients believed that stretching may also mitigate benign AEs. While our survey did not ask the rationale for participants’ responses, SMT is known to elicit muscle spindle activity [29], which may potentially contribute to benign AEs by influencing muscle contraction. Based on these responses, it is possible that clinicians and patients perceive soft tissue therapy or massage after SMT and stretching to minimize benign AEs potentially related to muscle spindle activation and its influence in muscular status. Participating clinicians also indicated that icing after SMT was likely to mitigate benign AEs. This is interesting considering patients did not share this belief. Icing or cryotherapy is often used for its anti-inflammatory and analgesic effects [30], thus it is possible that clinicians may perceive SMT creating an inflammatory response and minimizing this response with the use of ice. Further studies are warranted to clarify the underlying physiology of both benign AEs post-SMT and mitigation strategies.

Interestingly, specific mitigation strategies suggested to be applied before or after SMT changed the beliefs and perceptions of clinicians and patients related to their likelihood of being successful mitigators (Fig. 4). Previous studies have investigated the effect of the sequence of interventions in other health care fields, such as cardiac (heart rate and blood pressure) and training performance, and suggested that the order in which interventions are performed can influence the outcome [31–33]. Although no studies have investigated the effect of the sequence of interventions related to SMT, it is possible that, similarly to other health care fields, the order in which interventions and mitigators are performed can influence the frequency and/or severity of benign AEs post-SMT. This is an interesting topic and further prospective randomized studies will be conducted to determine the influence of mitigator sequence on benign AEs post-SMT.

### Strengths and limitations

This study reflects the perceptions and beliefs of those responding clinicians and patients at two chiropractic teaching institutes, therefore results should be interpreted with caution. It is possible that only patients with specific views and opinions towards benign AEs following SMT and mitigation strategies agreed to participate in the study. Additionally, given that this study was conducted at chiropractic teaching clinics, participating patients were mostly being treated by interns, who have less clinical experience and were not included in this study. However, we included participating clinicians with a range of clinical experience, as well as patients presenting with differing SMT experience, thereby reporting a variety of different opinions. In so doing, our results may be representative of clinicians and patients being seen in clinical practice. Additionally, although most supervising clinicians also practice in a community-based setting, it is likely that clinicians responded to the survey based on both of their clinical experience (teaching and community-based setting). Nevertheless, future studies including community-based practitioners and their patients in this specific should be conducted to investigate potential differences in beliefs and perceptions.

As previously mentioned, our survey was developed and validated specifically for this initial investigation and to answer specific questions in an efficient manner. While successful in terms of response rate, the survey did not enquire about the participants’ rationale for their responses. Future qualitative studies should be conducted to further explore clinicians’ and patients’ beliefs and perceptions of AEs and potential mitigating strategies.

Lastly, it is important to emphasize that our study focused on beliefs and perceptions of both clinicians and patients regarding strategies to mitigate benign AEs. Prospective studies will be conducted to investigate the effectiveness of these strategies so that clinical recommendations to mitigate benign AEs post-SMT can be included in best practice guidelines.

## Conclusion

Both clinicians and patients believe benign AEs occur post-SMT with pain/soreness, headache and stiffness being the most common benign AEs. However, clinicians and patients’ beliefs related to strategies to mitigate benign AEs post-SMT were not congruent, differing primarily in the application of icing and stretching. Aligning beliefs and perceptions of clinicians and patients related to mitigation strategies may contribute to reducing benign AEs post-SMT. Future randomized prospective studies will assess the clinical relevancy, effectiveness and influence of sequence of the mitigation strategies identified by clinicians and patients on reducing the frequency and severity of benign AEs post-SMT.

## Data Availability

The datasets used and analysed during the current study are available from the corresponding author on reasonable request.
